# Action of anti-TNF-α drugs on the progression of Alzheimer's disease: A case report

**DOI:** 10.1590/1980-57642015DN92000015

**Published:** 2015

**Authors:** Carlos Henrique Ferreira Camargo, Filipe Fernandes Justus, Giuliano Retzlaff, Marcelo Rezende Young Blood, Marcelo Derbli Schafranski

**Affiliations:** 1 Neurology Service, Medicine Department, Hospital Universitário – State University of Ponta Grossa, Ponta Grossa, Brazil.; 2 Rheumatology Service, Medicine Department, Hospital Universitário – State University of Ponta Grossa, Ponta Grossa, Brazil.

**Keywords:** TNF-α, Alzheimer's disease, rheumatoid arthritis

## Abstract

The aim of this study was to describe a clinical case of a patient with Alzheimer's disease (AD) in use of an anti-TNF-α agent for rheumatoid arthritis (RA). The patient reported is an 81-year-old Caucasian man and retired teacher, diagnosed with RA in 2008 and AD in 2011. Treatment with donepezil was started in 2011 and the use of etanercept introduced in 2012. He was previously treated with adalimumab in 2010 for 18 months. In 2013, the subject was engaged in a clinical trial to assess a complementary non-pharmacological approach for AD, presenting significant cognitive improvement during the follow-up period. We propose the hypothesis of a synergistic effect of anti-TNF-α medication used for the treatment of RA as the cause of the improvement in cognitive response observed. These findings could suggest a possible use of this drug class in the therapeutic management of AD.

## Abstract (pt)

O objetivo deste estudo foi descrever um caso de um paciente com doença de Alzheimer (DA) simultaneamente sob uso de medicação anti-TNF-α para artrite reumatoide (AR). Paciente masculino, 81 anos, caucasiano, professor aposentado. Diagnosticado com AR em 2008 e DA em 2011. O tratamento com donepezila foi iniciado em 2011, e o uso de etanercept instituído em 2012. O paciente foi tratado anteriormente com adalimumab em 2010 durante 18 meses. Em 2013, participou de um ensaio clínico visando a avaliar uma estratégia não farmacológica complementar para a DA, apresentando melhora cognitiva significativa ao longo do período de seguimento. Sugere-se a hipótese de um efeito sinérgico do agente anti-TNF-α utilizado para tratamento da AR como causa da melhor resposta cognitiva do paciente, indicando possível utilidade dessa classe medicamentosa na abordagem terapêutica da DA.

## INTRODUCTION

Among the numerous forms of dementia, Alzheimer's disease (AD) is the most common.^1^ Pathophysiologically, AD is associated with the accumulation of β-amyloid peptide aggregates in the brain cortex and hippocampus, which leads to the development of local inflammatory response, contributing to neuronal destruction and tissue atrophy.^2^ Several cytokines are possibly involved in the generation and maintenance of this inflammatory reaction, such as the Tumor Necrosis Factor-alpha (TNFα) and interferon-gamma (IFNγ), which can also mediate the production and deposition of β-amyloid aggregates.^3^ TNFα plays a major role in the progression of systemic inflammatory response, pathologically related to other commonly observed conditions in clinical practice, such as rheumatoid arthritis (RA).^4^ There is the possibility that high levels of pro-inflammatory cytokines may inhibit the phagocytosis of β-amyloid aggregates in the nervous tissue performed by the microglia.^5^

Since the early 1990s, an antagonistic relationship between AD and RA has been suggested, given that a significantly lower prevalence of AD was found in patients under long-term treatment with non-steroidal anti-inflammatory agents for RA.^6^ Pharmacologic management of RA was linked to delayed onset of cognitive symptoms in patients genetically predisposed to AD.^7,8^ Currently, genetic polymorphisms associated with the codifying gene of TNF-α have been linked to the risk of developing RA^8,9^ and also AD.^10^ TNF-α is a cytokine that performs homeostatic and pathophysiologic functions in multiple systems, including the central nervous system. Neurons, glial cells, macrophages and other cells of the immune system produce this factor under extremely variable stimuli. Its broad spectrum of action, not only restricted to inflammatory phenomena, makes the therapeutic approaches involving TNF-α highly relevant.^11,12^ Moreover, there are genetic characteristics that can explain an improved response to the treatment of RA with anti-TNF-α drugs,^13^ which raises the possibility of applying the same concept to AD.^10^

The main anti-TNF-α agents available are infliximab, adalimumab and etanercept, developed after the discovery of the key role played by TNF-α in the systemic inflammatory process, serving as useful alternative therapeutic approaches for several chronic inflammatory conditions, with RA figuring as the prototype disease.^14^ Despite the conflicting evidence on the theme, great attention has been dedicated to anti-TNF therapies for neurodegenerative diseases over the past decade, especially for AD.

The goal of this study was to assess the cognitive changes observed in a patient with AD and RA in use of an anti-TNF-α agent.

## CASE REPORT

The patient, an 81-year-old Caucasian married, holding a Bachelor's degree, and a retired teacher, started to present behavioral changes approximately five years earlier: he left the home several times without informing his destination and became blunted, isolating himself in the bedroom for hours at a time. The subject had always had good memory, however, from this date on, he manifested progressive memory deficits, becoming repetitive and also having major difficulty remembering more recent facts, even those related to his daily activities and personal life. Neurologic exam disclosed no other deficits besides cognitive dysfunction. Over time, the patient presented marked mood swings, alternating between periods of somnolence and agitation. Likewise, time and space disorientations emerged, associated with increased memory impairment. The Mini-Mental State Examination (MMSE)^15^ demonstrated temporospatial disorientation and inability to evoke two out of three words (score of 24/30). HIV, VDRL, vitamin B12, renal and hepatic function exams were all normal. Cranial computed tomography scan showed global cerebral volume loss, with no signs of atrophy in the medial temporal regions. Magnetic nuclear resonance was not performed at the time of diagnosis. Based on the results obtained, the patient was diagnosed with AD in 2011, and appropriate treatment with donepezil was prescribed, initially at 5 mg per day, then 10 mg, with good tolerance. Later, sertraline 50 mg per day was added to the scheme, followed by 25 mg of quetiapine during the night due to periods of nocturnal agitation. To date, The worst decompensation episode occurred owing to urinary tract infections and refractory joint pain related to RA.

The subject was also diagnosed with RA in 2008. At the beginning of the disease, he was under treatment with methotrexate, 15 mg a week, and prednisolone, 20 mg a day. Methotrexate was later substituted by leflunomide, 20 mg per day, and the corticosteroid was gradually withdrawn. The patient presented significant clinical improvement, persisting for approximately four months. The pain episodes became more frequent and intense, leading to the use of adalimumab (Humira™ - September, 2010) initially at 40 mg a week with dosage reduction after two months to 40 mg every fifteen days. With anti-TNF-α treatment, the patient reported substantial improvement in the symptoms. Appropriate disease control was observed for eleven months, when the subject again presented severe morning stiffness and diffuse pain. Corticosteroid therapy was reintroduced, with administration of 5 mg a day of prednisolone. Six months later, the patient was still reporting joint pain and persistent morning stiffness. Thus, it was decided to modify the anti-TNF-α strategy through the use of etanercept (Enbrel™ - July 2012) at 50 mg a week. After therapeutic modification, there was significant improvement in the symptoms related to RA, currently maintained for fifteen months.

**Figure 1:**
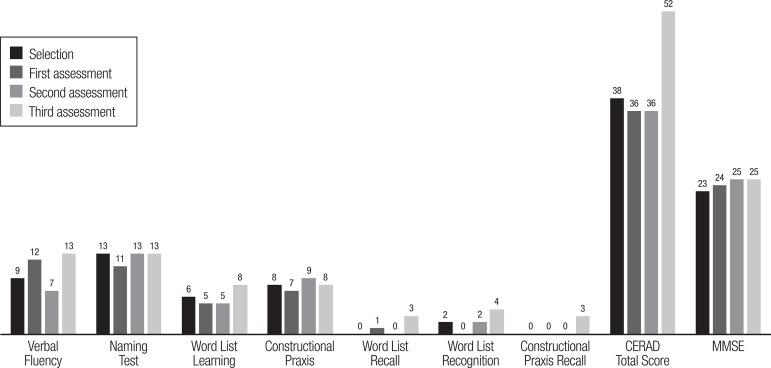
Subject's cognitive outcomes on bimonthly assessments during a 6-month follow-up period (CERAD and MMSE). CERAD. Consortium to Establish a Registry for Alzheimer’s Disease; MMSE, Mini-Mental State Examination. Assessments performed bimonthly since July 2013.

In July 2013, the patient was engaged in a controlled trial to evaluate the effectiveness of Reality Orientation (RO) therapy in the treatment of AD.^16^ In this study, the subject was selected as part of the group submitted to the complementary treatment, participating in weekly individual sessions of RO for six months. The Consortium to Establish a Registry for Alzheimer's Disease (CERAD) Neuropsychologic battery^17^ the Mini-Mental Status Examination (MMSE)^15^ and the Clock Drawing Test (CDT)^18^ were used as outcome assessment tools. The patient showed significant cognitive improvement during the study (Figure 1).

## DISCUSSION

The amyloid plaques in AD are closely related to microglia cells and activated astrocytes, cells involved in the liberation of pro-inflammatory factors and cytokines, such as TNF-α.^19^ TNF-α is also associated with the increased accumulation of β-amyloid peptide aggregates in the central nervous system and the induction of neuronal death.^11^ Furthermore, the identification of high levels of specific cytokines in the cerebrospinal fluid and peripheral blood of subjects with AD, as well as elevated production of these components in the central nervous system,^20^ has made the subject of anti-TNF strategy for the treatment of neurodegenerative diseases a very attractive field in recent years. Nevertheless, certain findings contradict this tendency, suggesting that the absence of influence of TNF in the central nervous system may actually aggravate the pathophysiological scenario of AD.^21,22^

In those conditions that have formal therapeutic indication of the drugs, TNF-α inhibitors are applied peripherally. Although it has been established that these medications are unable to cross the blood-brain barrier under these conditions,^23^ a pre-clinical trial observed, for the first time, a reduction in the toxic effects induced by β-amyloid aggregates associated with decreased levels of TNF-α in the central nervous system after the administration of peripheral etanercept in animal models.^24^ These results contradict the findings of earlier trials involving the use of the same drug.^25^ Considering the pharmacologic characteristics of these agents and experience gained in the treatment of neurologic disorders, several studies have been conducted since 2004 addressing the perispinal injection of anti-TNF medications for the treatment of AD.^12,26^ This research model yielded promising outcomes, evidenced by quick and sustained cognitive improvements in the individuals treated.^12^ However, further trials need to be conducted to fill some gaps in knowledge, such as the physiologic repercussions of fully inhibitingTNF-α effects in the central nervous system and the possibility of selective inhibition of these cytokine functions.^11^

In the case report, the favorable progression of the arthritic symptoms after starting etanercept therapy and the marked cognitive response of the subject in comparison to the other participants in the controlled trial reported,^16^ suggest that concomitant use of an anti- TNF-α agent could have contributed to the superior cognitive performance of the patient. Analyzing the MMSE score progression during the trial, the patient's score increased by 2 points, a significantly greater improvement than the average for the other participants in the group (1.43 point).^16^ In a similar study conducted by Onder et al.,^27^ during a 25-week follow-up period, the mean improvement in MMSE score in the group submitted to regular RO sessions was 0.2 point (SE 0.4), much lower than that observed in this case report. Another trial with similar characteristics also showed less marked improvement compared to that found in the present patient, reporting a mean improvement of 0.71 points after a 6-month follow-up.^28^

The patient's performance on the CERAD neuropsychological battery,^17^ an innovative methodological aspect of the trial, used as part of the cognitive assessment during follow-up, also yielded interesting results.^16^ By the end of the follow-up, the improvement in CERAD total score observed for the subject was +14 points, more than twice the average improvement of the other participants in the treatment group (6.43 points).^16^ Although there are no similar studies against which these results can be compared and no other significant disparities in specific items of the battery were found between the patient and the rest of the group, these findings may corroborate the hypothesis of a positive contribution of anti-TNF-α agents in this individual's clinical outcomes.

Despite the inability to cross the blood-brain barrier, there are several mechanisms by which the inflammatory response can provoke neurotoxicity without direct release of cytokines into the central nervous system.^20^ Likewise, inflammatory signals can propagate through the blood-brain barrier via communication between peripheral and central immune cells.^20,29,30^ Another relevant aspect that corroborates this hypothesis is that the adequate response to the anti-TNF-α therapy observed in the patient described can be derived from a favorable genotype to this kind of intervention.^8,9,13^ This could also translate to other benefits from the suppression of TNF-α activity, observed in this case as the marked cognitive response obtained after engagement in a cognitive stimulation strategy. This is especially pertinent in view of recent findings observed in the pre-clinical trial using animal models, which suggested for the first time that the peripheral administration of anti-TNF-α agents has therapeutic potential in countering the deleterious effects of neuroinflammation in AD.^24^

This case report illustrates the possible action of anti-TNF-α drugs in the control of AD. It is hoped that the promising studies currently being conducted can translate to novel treatment approaches for AD, confirming the initial outcomes.
